# Colour variation in red grapevines (*Vitis vinifera *L.): genomic organisation, expression of flavonoid 3'-hydroxylase, flavonoid 3',5'-hydroxylase genes and related metabolite profiling of red cyanidin-/blue delphinidin-based anthocyanins in berry skin

**DOI:** 10.1186/1471-2164-7-12

**Published:** 2006-01-24

**Authors:** Simone D Castellarin, Gabriele Di Gaspero, Raffaella Marconi, Alberto Nonis, Enrico Peterlunger, Sophie Paillard, Anne-Francoise Adam-Blondon, Raffaele Testolin

**Affiliations:** 1 Dipartimento di Scienze Agrarie e Ambientali, University of Udine, via delle Scienze 208, 33100 Udine, Italy; 2 UMR de Génomique Végétale, INRA-CNRS-UEVE, 2, Rue Gaston Crémieux, CP5708, 91057 Evry Cedex, France; 3 UMR118 INRA-AgroCampus Rennes, Amélioration des Plantes et Biotechnologies Végétales, Domaine de la Motte, BP 35327, 35653 Le Rheu Cedex, France

## Abstract

**Background:**

Structural genes of the phenyl-propanoid pathway which encode flavonoid 3'- and 3',5'-hydroxylases (F3'H and F3'5'H) have long been invoked to explain the biosynthesis of cyanidin- and delphinidin-based anthocyanin pigments in the so-called red cultivars of grapevine. The relative proportion of the two types of anthocyanins is largely under genetic control and determines the colour variation among red/purple/blue berry grape varieties and their corresponding wines.

**Results:**

Gene fragments of *VvF3'H *and *VvF3'5'H*, that were isolated from *Vitis vinifera *'Cabernet Sauvignon' using degenerate primers designed on plant homologous genes, translated into 313 and 239 amino acid protein fragments, respectively, with up to 76% and 82% identity to plant CYP75 cytochrome P450 monooxygenases. Putative function was assigned on the basis of sequence homology, expression profiling and its correlation with metabolite accumulation at ten different ripening stages. At the onset of colour transition, transcriptional induction of *VvF3'H *and *VvF3'5'H *was temporally coordinated with the beginning of anthocyanin biosynthesis, the expression being 2-fold and 50-fold higher, respectively, in red berries versus green berries. The peak of *VvF3'5'H *expression was observed two weeks later concomitantly with the increase of the ratio of delphinidin-/cyanidin-derivatives. The analysis of structural genomics revealed that two copies of *VvF3'H *are physically linked on linkage group no. 17 and several copies of *VvF3'5'H *are tightly clustered and embedded into a segmental duplication on linkage group no. 6, unveiling a high complexity when compared to other plant flavonoid hydroxylase genes known so far, mostly in ornamentals.

**Conclusion:**

We have shown that genes encoding flavonoid 3'- and 3',5'-hydroxylases are expressed in any tissues of the grape plant that accumulate flavonoids and, particularly, in skin of ripening red berries that synthesise mostly anthocyanins. The correlation between transcript profiles and the kinetics of accumulation of red/cyanidin- and blue/delphinidin-based anthocyanins indicated that *VvF3'H *and *VvF3'5'H *expression is consistent with the chromatic evolution of ripening bunches. Local physical maps constructed around the *VvF3'H *and *VvF3'5'H *loci should help facilitate the identification of the regulatory elements of each isoform and the future manipulation of grapevine and wine colour through agronomical, environmental and biotechnological tools.

## Background

Vacuolar accumulation of anthocyanins in berry skin is responsible for the pigmentation of red/blue grapevine (*Vitis vinifera*) cultivars. Anthocyanin biosynthesis is developmentally triggered at the onset of berry ripening (8–10 weeks after blooming) and lasts till harvest [1]. Expression profiling of structural genes and transcription factors has claimed a major role for the gene *UFGT *encoding a UDP-glucose:flavonoid 3-O-glucosyltransferase and its regulatory *Myb*-type gene *VvMybA *in conveying the flux of flavonoid intermediates towards the synthesis of anthocyanins [2,3]. The enzyme UFGT is synthesised since *véraison *under the control of *VvMybA *and catalyses the 3'-glycosidation of cyanidin- and delphinidin-based anthocyanidins [4]. The resulting stably coloured anthocyanins may undergo methylation upon the activity of methyl-transferases that convert cyanidin into peonidin and delphinidin into petunidin or malvidin, similarly to how it occurs in other anthocyanin-producing species [5,6].

Cyanidin- and delphinidin-derived anthocyanins diverge from each other regarding the number of the substituted groups that are found on the B-ring of the flavonoid skeleton (either two or three, respectively) and have their chromatic properties affected accordingly. Cyanidin-based anthocyanins exhibit a reddish colour whereas delphinidin-based anthocyanins are purple to blue. They jointly determine the tonality of red berry grape varieties and their corresponding wines. The relative proportion of the five anthocyanins is largely under genetic control and unique to each cultivar [7,8]. Sunlight exposition and water deficit have been shown to modulate to some extent the anthocyanin profile [9,10].

In plant species, the biosynthesis of cyanidin- and delphinidin-type precursors is driven, upstream of the enzyme UFGT, by the activity of two flavonoid 3'- and 3',5'-hydroxylases (F3'H and F3',5'H) that add either a single hydroxyl group at the 3' position or two hydroxyl groups at the 3' and 5' positions to dihydrokaempferol and/or dihydroquercetin. Once converted into 3'-dihydroquercetin or 3',5'-dihydromyricetin, these intermediates flow through common downstream enzymes to form di-substituted and tri-substituted anthocyanins when the gene *UFGT *is expressed and to form other polyphenols (flavonols, leucoanthocyanins, catechins, proanthocyanins, tannins) at different developmental stages. F3'H and F3',5'H genes code for CYP75 cytochrome P450 hydroxylases that have been well characterised in the ornamental plants *Antirrhinum majus *[11], *Campanula medium *[12], *Dianthus caryophyllus *[13], *Gentiana triflora *[14], *Ipomoea purpurea*, [15], *Petunia hybrida *[16], *Phalaenopsis *[17] and *Vinca major *[18]. In all these species, F3'H and F3',5'H are found as low/medium copy number genes and have been genetically manipulated to produce novel flower pigmentation. In grapevine, only tentative consensus (TC) sequences for F3'H and F3',5'H genes are available in the TIGR grape gene database . TCs were built based on partially overlapping ESTs from cDNA libraries obtained from different genotypes and tissues. This makes it unfeasible to predict or rule out the presence of different isogenes.

This paper reports (1) the isolation of flavonoid 3'- and 3',5'-hydroxylase gene members in grapevine via sequence homology, (2) the assessment of their chromosomal location by genetic and physical mapping, (3) their transcript profiles in different tissues, (4) the time-course of expression in berry skin of the red cultivar 'Merlot' from pre-*véraison *to harvest compared to the curves of accumulation of anthocyanins, catechins and total polyphenols.

## Results and discussion

### Isolation of flavonoid 3'- and 3',5'-hydroxylase genes in grapevine

Gene isolation was accomplished via sequence homology with the help of newly designed degenerate primers. PCR bands that were amplified from genomic DNA of *Vitis vinifera *'Cabernet Sauvignon' were longer than predicted on the basis of plant flavonoid hydroxylase transcripts (Table 1 and 2).

**Table 1:** List of the PCR primers used.

**Target DNA**	**Name**	**5'→3' sequence^a^**
Plant F3'H	F3'H_S	CCIGTIAANYTNGGNCARYTIBTIAA
Plant F3'H	F3'H_AS	GCICKYTGIARIGTNARNCCRTA
Plant F3'5'H	F3'5'H_S	ATGCTGACNTAYGCIATGGCNAAYATG
Plant F3'5'H	F3'5'H_AS	CGAARTCRTTICCYCKNGGNTCDATYTT
Grape F3'H	VvF3'H_S	ATTCGCCACCCTGAAATGAT
Grape F3'H	VvF3'H-indel_S	CAACAAGAGCTGGACGCAGT
Grape F3'H	VvF3'H_AS	AGCCGTTGATCTCACAGCTC
Grape F3'5'H	Vv3'5'H-1A_S	GAAGTTCGACTGGTTATTAACAAAGAT
Grape F3'5'H	VvF3'5'H-1A_AS	AGGAGGAGTGCTTTAATGTTGGTA
Grape F3'5'H	Vv3'5'H-2A_S	AAGAAGTTCGACAAGTTATTGACAAG
Grape F3'5'H	Vv3'5'H-2A_AS	AGCGCCTTAATGTTGGTAAGG
Grape F3'H	VvF3'H-intron_S	CGTTGATCTCGCTGAAGGAT
Grape F3'H	VvF3'H-intron_AS	ATCATTTCAGGGTGGCGAAT
Grape F3'5'H	Vv3'5'H-1A-intron_S	GCTCACTATTACCAACATTAAAGCA
Grape F3'5'H	Vv3'5'H-2A-intron_S	AAGCTCACCCTTACCAACATTAAG
Grape F3'5'H	Vv3'5'H-intron_AS	GGCAAGTCAGACTCCACGAG
BES38C24-FM	38C24-FM_S	TGGCCTTCATTTTGGTTAGC
BES38C24-FM	38C24-FM_AS	TCATCACTCAGGGCACAAAA
BES38C24-RM	38C24-RM_S	TTACCGCCCTTGTCCTTGTA
BES38C24-RM	38C24-RM_AS	GCGTATTGATCATCCCTTGG
BES17K4-FM	17K4-FM_S	CCAATATGTGGAGGAGGAACA
BES17K4-FM	17K4-FM_AS	CATCCAGGTTGTGAAGGACA
BES9C17-FM	9C17-FM_S	AGGAGGCCTATGCATCAGAA
BES9C17-FM	9C17-FM_AS	GGAGCACCATTAAAGGCAAA
BES40A22-FM	40A22-FM_S	CTAATGGGCATCAGGTTTCG
BES40A22-FM	40A22-FM_AS	CGTGCATATCATTTTCTTCCAA
BES5A23-FM	5A23-FM_S	TTCAAGAAGTGGAGGCTGCT
BES5A23-FM	5A23-FM_AS	AGCACTGACTGCCTGATCCT

^a ^Degenerate IUB code: I = inosine; R = A or G; M = A or C; W = A or T; Y = C or T; S = C or G; D = A, G or T; N, A, C, G or T.

**Table 2:** Primers pairs and PCR conditions used for the amplification of grapevine flavonoid hydroxylase genes.

**Code**	**Sense primer**	**Anti-sense primer**	**Expected size (bp)**	**Annealing temp. (°C)**
F3'H	F3'H_S	F3'H_AS	825*	60
F3'5'H	F3'5'H_S	F3'5'H_AS	1020*	56
F3	VvF3'H_S	VvF3'H_AS	196	58
F3-1	VvF3'H-indel_S	VvF3'H_AS	166	58
F35-1	Vv3'5'H-1A_S	VvF3'5'H-1A_AS	156	58
F35-2	Vv3'5'H-2A_S	Vv3'5'H-2A_AS	152	65
F3int	VvF3'H-intron_S	VvF3'H-intron_AS	255	58
F35-1int	Vv3'5'H-1A-intron_S	Vv3'5'H-intron_AS	589	58
F35-2int	Vv3'5'H-2A-intron_S	Vv3'5'H-intron_AS	593	58
38C24-FM	38C24-FM_S	38C24-FM_AS	312	58
38C24-RM	38C24-RM_S	38C24-RM_AS	330	58
17K14-FM	17K4-FM_S	17K4-FM_AS	327	58
9C17-FM	9C17-FM_S	9C17-FM_AS	358	58
40A22-FM	40A22-FM_S	40A22-FM_AS	327	58
5A23-FM	5A23-FM_S	5A23-FM_AS	218	54

* in absence of introns.

**Figure 1:**
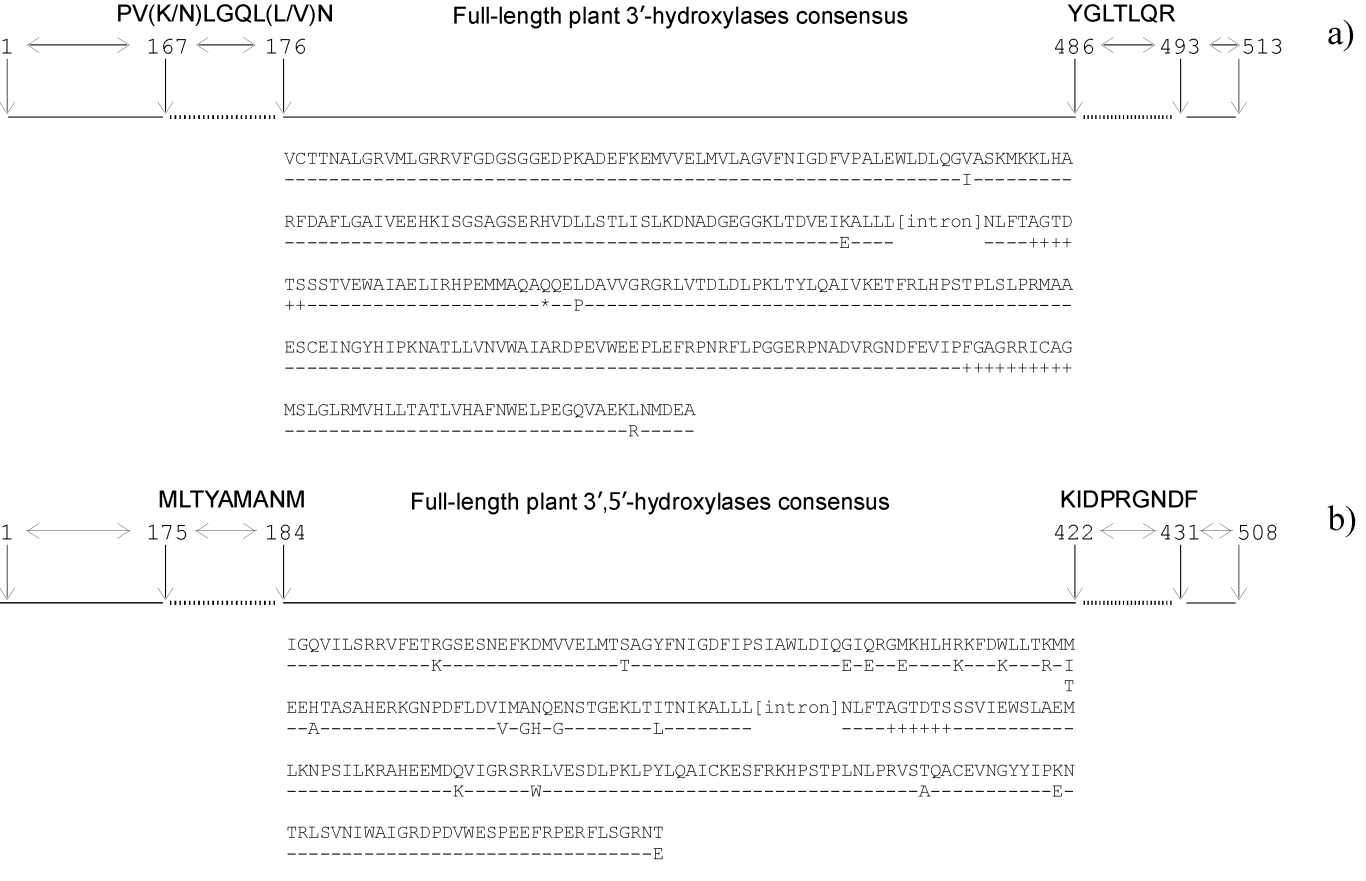
**Grapevine flavonoid hydroxylase gene fragments isolated from 'Cabernet Sauvignon' genomic DNA**. The position of the conserved sites among plant flavonoid hydroxylases, on which the degenerate primers were designed, and the region of the gene fragments sequenced in grapevine are referenced to the gene *Ht1 *of *Petunia *× hybrida [GenBank:AF155332] for the flavonoid 3'-hydroxylase (a) and to the *Petunia *× hybrida GenBank:Z22544 for the flavonoid 3',5'-hydroxylase (b) [41]. The consensus amino acid sequences of grapevine *VvF3'H *and *VvF3'5'H *gene fragments are shown along with the position of the intron and (*line below *the sequence) the variable sites among all the sequenced fragments. The *symbol *'-' stands for invariable position, '+' underlines two functional domains invariantly found in plant flavonoid hydroxylases, '*' indicates the site of the 23-bp deletion in the *VvF3'H-2 *sequence.

Five different sequences were isolated with the F3'H degenerate primers following SSCP analysis and were given the designation *VvF3'H-1a*, *-1b*, *-1c*, *-1d*, and *VvF3'H-2 *[GenBank:DQ298196-DQ298200]. Once the primer sites had been removed, the sequences *VvF3'H-1a *to *-1d *were 1,034 bp long with a 95 bp intron; *VvF3'H-2 *was 1,011 bp long with a 95 bp intron. Sizing of genomic amplicons performed by capillary electrophoresis confirmed that the two classes of amplicons diverge from each other for a 23-bp indel. Sequence identity was between 99.8 and 99.9% (99.7–99.8% for the coding region) among the sequences *VvF3'H-1*. The presence of mutations introduced by *Taq *polymerase during the PCR process and immortalised through the cloning of single PCR-amplified molecules could not be ruled out as the origin of differences among the four *VvF3'H-1 *sequences. The sequences *VvF3'H-1a *to *-1d *translated into protein fragments of 313 amino acids with 311 positions conserved among the sequences when pair-wise comparisons were performed (Figure 1). *VvF3'H-2 *contained a frame shift mutation due to a 23-bp deletion in the coding region. Function was putatively assigned to *VvF3'H-1 *based on its highest identity match in GenBank at the protein level (76% identity, E = 8e^-134^) that corresponded to a flavonoid 3'-hydroxylase of *Petunia *× hybrida [GenBank:AAD56282]. The nucleotide coding sequence of *VvF3'H-1 *matched the grapevine tentative consensus sequence TC42042 that has been predicted from three partially overlapping ESTs sequenced in 'Cabernet Sauvignon' and 'Chardonnay' (TIGR grape gene index). The *VvF3'H-1 *protein fragments spanned the 1–245 amino acid region of TC42042 and extended it on the 5' end of the gene by 67 amino acids. Amino acid sequence identity between the four *VvF3'H-1 *protein fragments and TC42042 was 95–96%.

**Figure 2:**
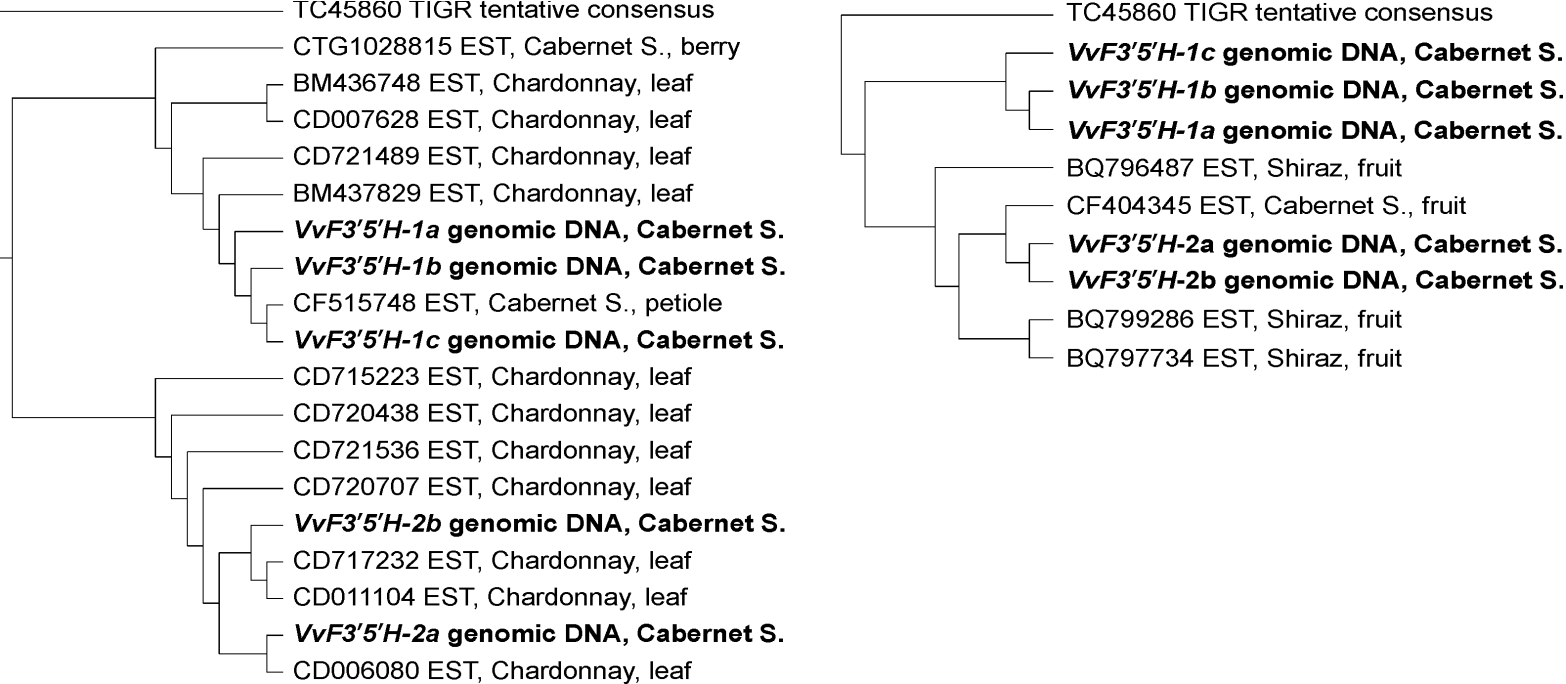
**N-J clustering of *Vitis vinifera *flavonoid 3',5'-hydroxylase genes**. Nucleotide sequences *VvF3'5'H-1a*, *-1b*, *-1c*, *-2a *and *-2b *from 'Cabernet Sauvignon' (in *bold*) were aligned along with ESTs singletons and the resulting tentative consensus (TC45860) for grapevine F3'5'H held at the TIGR grape gene index. ESTs covered both the 5'-end of the *VvF3'5'H *gene fragments (*left*) and the 3'-end (*right*). The tentative consensus has been forced as an outgroup. For each EST, the GenBank ID is reported along with the source genotype and tissue.

The degenerate primers targeting F3'5'H yielded five sequences that were given the designation *VvF3'5'H-1a*, *-1b*, *-1c*, *VvF3'5'H-2a*, *-2b *[GenBank:DQ298201-DQ298205]. Once the degenerate primer sites had been removed, the sequences *VvF3'5'H-1a*, *-1b *and *-1c *were 1,120 bp long with a 403 bp intron; *VvF3'5'H-2a *and *VvF3'5'H-2b *were 1,122 bp long with a 405 bp intron. The translated coding region matched several plant F3'5'H genes in GenBank. *VvF3'5'H-1a*, *-1b *and *-1c *shared the highest identity score at the amino acid level with the accession AAP31058 of *Gossypium hirsutum *(81%, E = e^-111^) as well as *VvF3'5'H-2a *and *-2b *did with the accession AAM51564 of *Glycine max *(82%, E = e^-112^). Translated sequences of *VvF3'5'H-1a *and *VvF3'5'H-1b *diverged from each other for two out of 239 amino acids. The same occurred between the sequences *VvF3'5'H-2a *and *VvF3'5'H-2b *(Figure 1). The sequences *VvF3'5'H-1a *and *VvF3'5'H-1b *had 93–94% identity at the amino acid level with *VvF3'5'H-2a *and *VvF3'5'H-2b*. All sequences matched the tentative consensus TC45860 obtained from 19 ESTs at the TIGR grape gene index. The 'Cabernet Sauvignon' gene fragments identified in this work spanned the 187–424 amino acid region of TC45860 over a predicted ORF of 508 amino acids. The sequences *VvF3'5'H-1a *and *VvF3'5'H-1b *had 97% and 98% amino acid identity, respectively, with TC45860; *VvF3'5'H-2a *and *VvF3'5'H-2b *peptide sequences had 95% identity with TC45860. The *VvF3'5'H *gene fragments partially overlapped also with a EST (ID CTG1028815) of a 'Cabernet Sauvignon' berry cDNA library already analysed by MPSS . In order to predict how many functional copies of the gene *VvF3'5'H *are present in the grape genome we looked at the number of different ESTs available at the grape gene databases. Twelve EST singletons held at the TIGR and the EST CTG1028815 spanned the 5' end of the *VvF3'5'H *gene fragments whilst four ESTs were alignable to the 3' end. All 17 ESTs, irrespective of their tissue-specific localisation and the source genotype, were aligned with the 'Cabernet Sauvignon' genomic sequences *VvF3'5'H-1a*, *-1b*, *-2a*, *-2b *(Figure 2). Six ESTs clustered with the 'Cabernet Sauvignon' *VvF3'5'H-1a *and *VvF3'5'H-1b *gene fragments. Those ESTs were detected in leaves of the white variety 'Chardonnay' and in petioles and berries of the red variety 'Cabernet Sauvignon'. Eleven ESTs grouped together with the 'Cabernet Sauvignon' *VvF3'5'H-2a *and *VvF3'5'H-2b *genomic sequences. The latter ESTs were present in various tissues (leaves and berries) of red ('Cabernet Sauvignon' and 'Syrah') and white ('Chardonnay') cultivars. Two major groups of F3'5'H transcripts including either *VvF3'5'H-1 *or *VvF3'5'H-2 *can be detected in different tissues of both red and white grapevines, suggesting that at least two genes encoding CYP75A cyt P450 hydroxylases are expressed in flavonoid-synthesizing tissues. Due to the putative presence of SNPs in ESTs from different cultivars, it was not possible to ascertain the number of functional copies belonging to each group, but together with the number of highly similar sequences obtained from 'Cabernet Sauvignon' genomic DNA, this pointed to the presence of multiple gene members coding for different isoforms of flavonoid hydroxylases in the grape genome.

**Figure 3:**
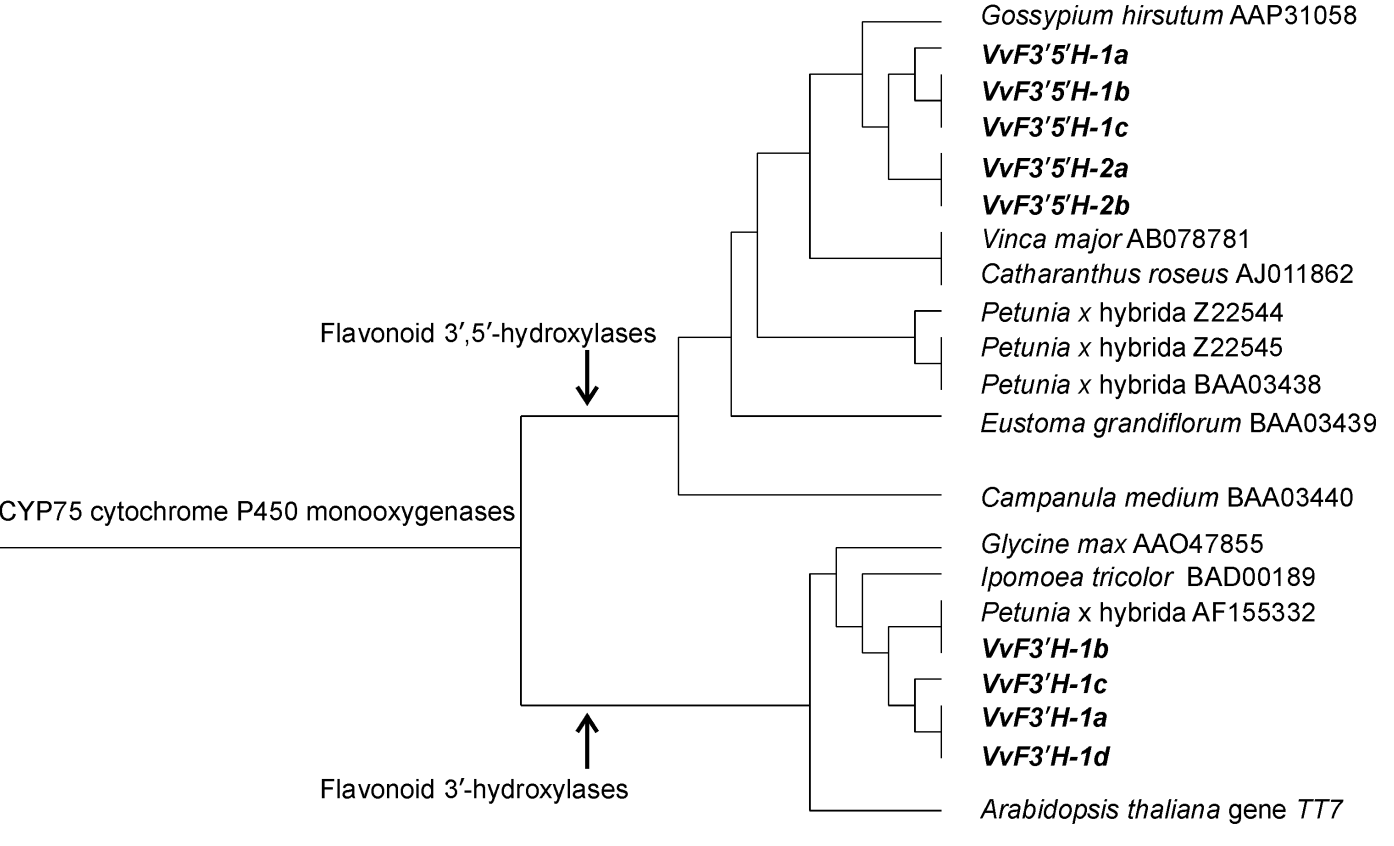
**The CYP75 clan of plant cyt P450s**. The branch containing the CYP75 family is focused on a Neighbor-Joining tree constructed using 670 plant cytochrome P450 monooxygenases. Flavonoid 3'-hydroxylases *VvF3'H-1a *to *-1d *and flavonoid 3',5'-hydroxylases *VvF3'5'H-1a *to *-2b *that were identified in grapevine are in *bold*. They split into two lower hierarchical branches that identified the CYP75B (flavonoid 3'-hydroxylases) and CYP75A (flavonoid 3',5'-hydroxylases) sub-families, respectively.

In order to test the phylogenetic relationship between grapevine hydroxylases and the multi-functional family of plant monooxygenases, a Neighbor-Joining tree was constructed using translated sequences of *VvF3'H*, *VvF3'5'H-1*, *VvF3'5'H-2 *and 670 protein entries of plant cyt P450 monooxygenases (Figure 3). *VvF3'H *and *VvF3'5'H *grouped solely with P450 proteins specifically annotated as flavonoid hydroxylases belonging to the family-specific branch of CYP75 monooxygenases. *VvF3'H *and *VvF3'5'H *split into two terminal branches, each one hosting either CYP75B or CYP75A monooxygenases. Although most plant P450s are of polyphyletic origin and some P450 families are lineage-specific [19], CYP75 P450s are ancient genes that existed even before the separation between gymnosperms and flowering plants [20]. The ancient origin of CYP75 monooxygenases is also supported by the finding that intron position of *VvF3'H *and *VvF3'5'H *was conserved when compared to other plant genes coding for flavonoid hydroxylases such as the *Arabidopsis *gene *TT7 *[21].

### Genetic mapping

**Figure 4:**
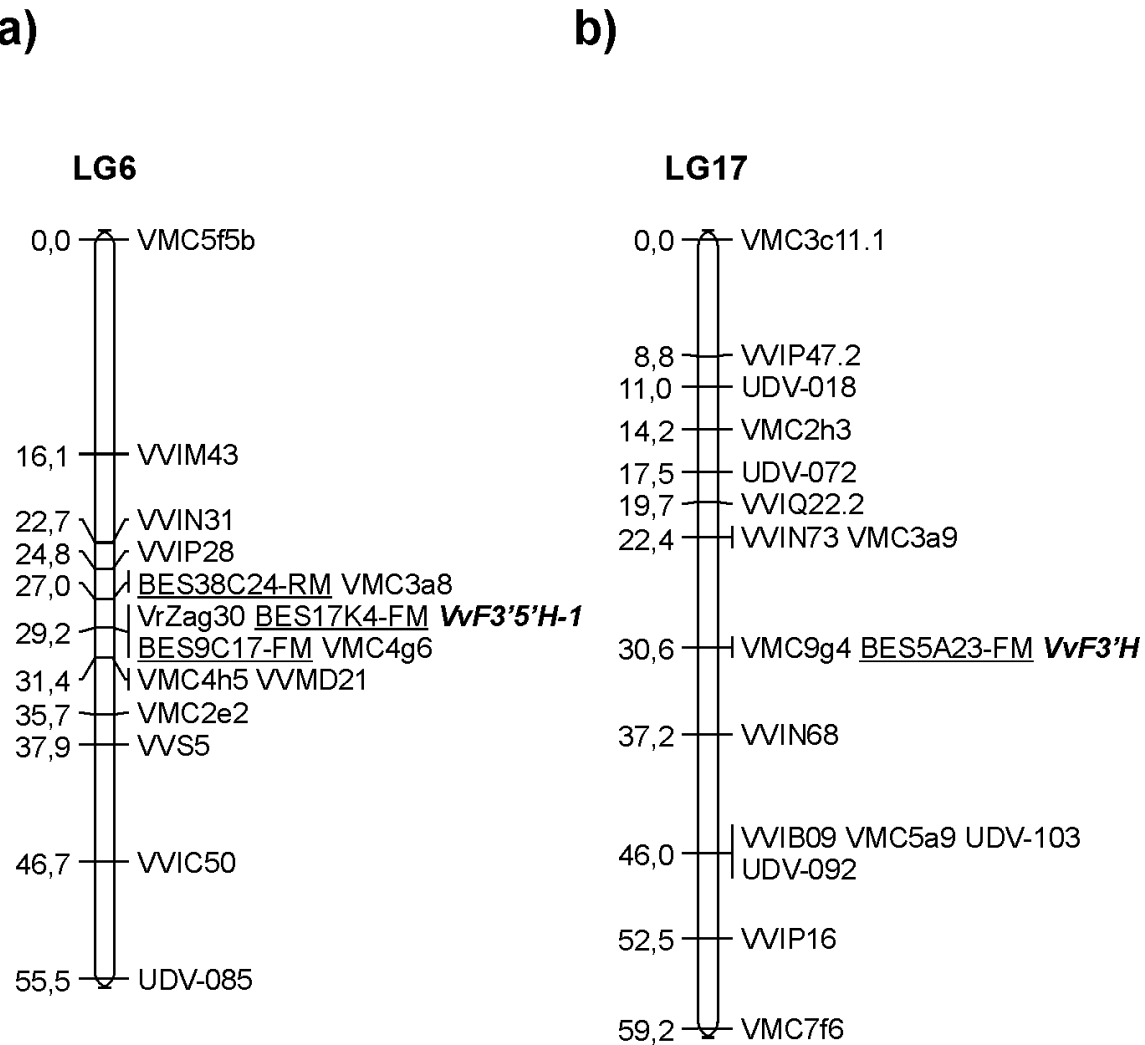
**Genetic map of 'Cabernet Sauvignon' LG 6 (a) and LG 17 (b) including the *VvF3'5'H *and *VvF3'H *loci**. *VvF3'H *and *VvF3'5'H *loci (in *bold*) were mapped with the use of the SSCP markers F3H and F35-1int, respectively. BES markers (*underlined*) were generated from the end-sequences of BAC clones positive for *VvF3'H *and *VvF3'5'H *and mapped using the SSCP technique. VMC, VVI, UDV, VVMD, VVS, and VrZag are microsatellite markers. *Numbers *on the left indicate the genetic distance between the markers expressed in centiMorgan (cM).

Genetic loci harbouring genes that code for flavonoid hydroxylases were mapped using the single strand conformational polymorphism (SSCP) analysis of F3'H and F3'5'H sequences in an F1 pseudo-testcross mapping population originated from the cross 'Cabernet Sauvignon' *Vitis *'breeding line 20/3'. The two genes segregated independently and were mapped to linkage groups LG17 and LG6, respectively (Figure 4). The linkage groups were annotated according to [22] and included also reference microsatellite markers and SSCP markers generated from BAC end-sequences (BES) of a 'Cabernet Sauvignon' BAC library. The locus containing the gene that codes for flavonoid 3'-hydroxylase was identified by linkage analysis following the segregation of the SSCP of two co-segregating bands amplified from the parent 'Cabernet Sauvignon' by means of the primer pair F3int (Table 2). The *VvF3'H *locus mapped to the middle of LG17 and co-segregated with the BES marker 5A23-FM and the microsatellite marker VMC9g4. The locus containing the genes that code for flavonoid 3',5'-hydroxylase was mapped by means of a polymorphic SSCP band amplified from the parent 'Cabernet Sauvignon' using the primer pair F35-1int. This marker mapped to the middle of LG6 and co-segregated with microsatellite marker VrZag30 and BES markers 9C17-FM and 17K4-FM (Figure 4). Other SSCP bands that were co-amplified by the same primer pair F35-1int could not be scored in the progeny due to faintness or homozygosis. SSCP marker F35-2int could not be mapped in the 'Cabernet Sauvignon' parental map, but co-segregated with VrZag30 and SSCP marker F35-1int in the male parent of the same cross (data not shown).

### Physical mapping and anchoring of BAC contigs to the genetic map

A total of 34 BAC clones containing the genes *VvF3'H *and *VvF3'5'H *were identified by screening a set of 18,432 BACs corresponding to a 6X genome coverage [23]. The primer pair F3, specific for grapevine flavonoid 3'-hydroxylase, detected 1.75 gene copies per haploid genome equivalent. Two bands of 196 bp and 173 bp, corresponding to the *VvF3'H-1 *and the *VvF3'H-2 *gene fragments, respectively, were co-amplified from all but two of the BACs, while the 196 bp band alone was amplified from the other two BAC clones. The genes coding for flavonoid 3',5'-hydroxylases were searched in the BAC library using the primer pairs F35-1 and F35-2 (Table 2). The primer pair F35-1, specific for the genes *VvF3'5'H-1*, identified five BAC clones. The primer pair F35-2, specific for *VvF3'5'H-2*, identified two different BAC clones. Twelve additional BAC clones were identified by both primer pairs. The number of positive BAC clones per haploid genome equivalent was on average 2.4 for the gene *VvF3'5'H-1 *and 2.75 for the gene *VvF3'5'H-2*. Local physical maps around the *VvF3'H *and *VvF3'5'H *loci were constructed using all positive BAC clones identified. Those BAC clones were used to query 2,035 BAC contigs resulting from a whole genome physical map. This map was assembled using FPC software [24] at a 10^-50 ^cut-off from 30,151 fingerprints of BAC clones derived from the same library and corresponding to an approximate coverage of 10X [S. Paillard, unpublished data].

**Figure 5:**
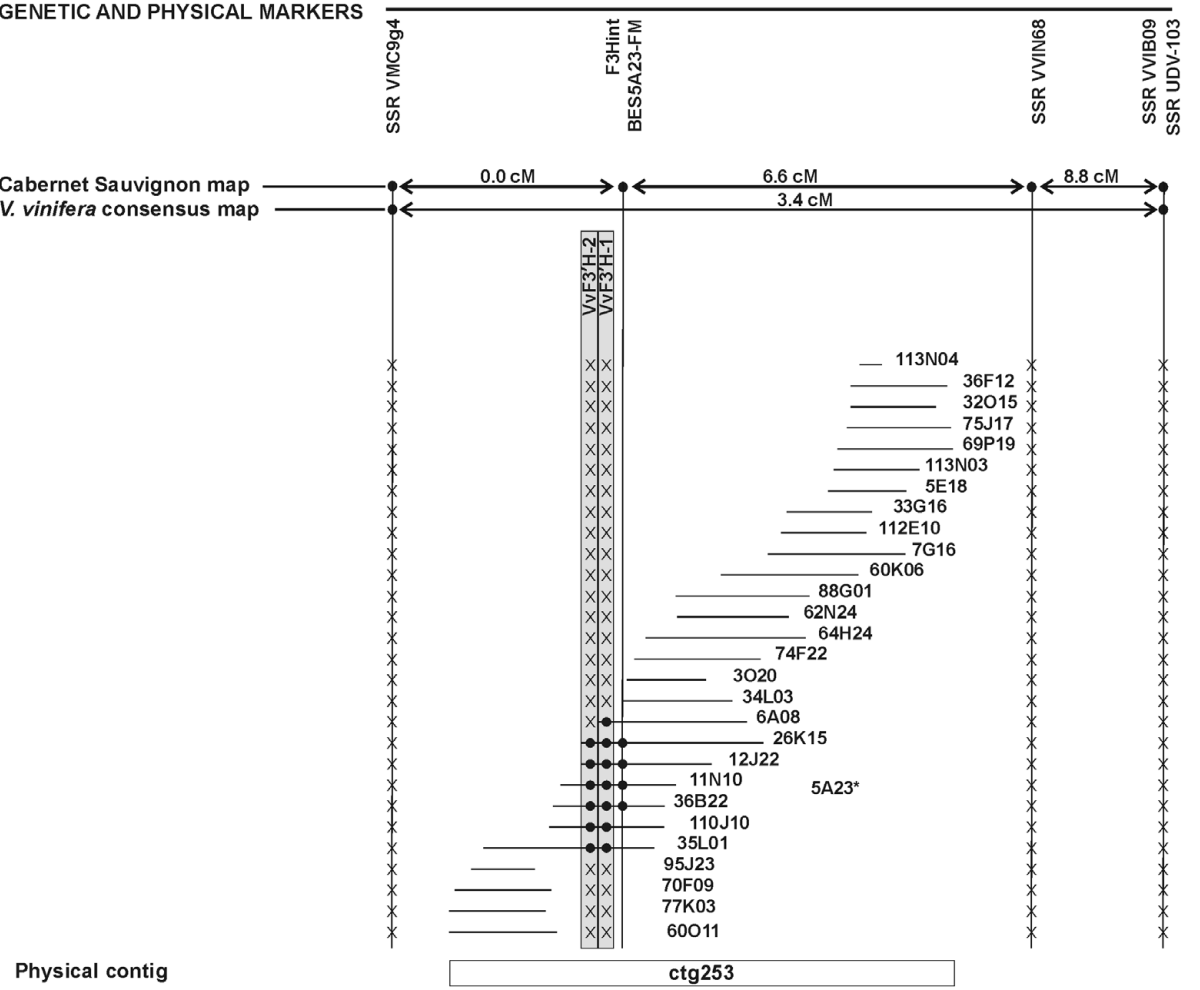
**Physical map of the *VvF3'H *locus**. Map of BAC contig ctg253 carrying the *VvF3'H-1 *and *VvF3'H-2 *genes and its anchorage to LG17 of 'Cabernet Sauvignon' and *V. vinifera *consensus genetic maps. BAC clone 5A23 bears the *VvF3'H-1 *isogene and was included after PCR anchoring onto the contig using marker BES5A23-FM. *Names *written in horizontal refer to BAC clones and contigs; those written in vertical refer to markers and genes. The orientation of the contig is arbitrary. Genetic and physical distances are not drawn to scale. The presence of a given marker on a BAC clone is highlighted with the symbol '●'. The symbol 'X' stands for lack of amplification of a given marker in the corresponding BAC clone. BAC clones missing any symbol at marker BES5A23-FM were not tested. Physical localisation of *VvF3'H-1 *and *VvF3'H-2 *loci is shown in grey *boxes*.

Two flavonoid 3'-hydroxylase genes, *VvF3'H-1 *and *VvF3'H-2*, were tightly linked in contig ctg253 that consisted of 28 BAC clones and approximately covered 700 kb (Figure 5). Six BAC clones contained both isogenes, whilst two BAC clones (6A08 and 5A23) contained only *VvF3'H-1*. BAC clone 5A23 was initially not included in the ctg253 following computational contig assembly because it did not meet the cut-off due to a low number of fingerprint bands. However, it was later placed in the contig upon PCR anchorage to other BAC clones using the BES-derived primers (BES5A23-FM). The same marker BES5A23-FM was used to anchor contig ctg253 to the 'Cabernet Sauvignon' genetic map. The marker based on the SSCP of the *VvF3'H *gene fragments and the BES-derived marker co-segregated at the level of resolution of the genetic map.

**Figure 6:**
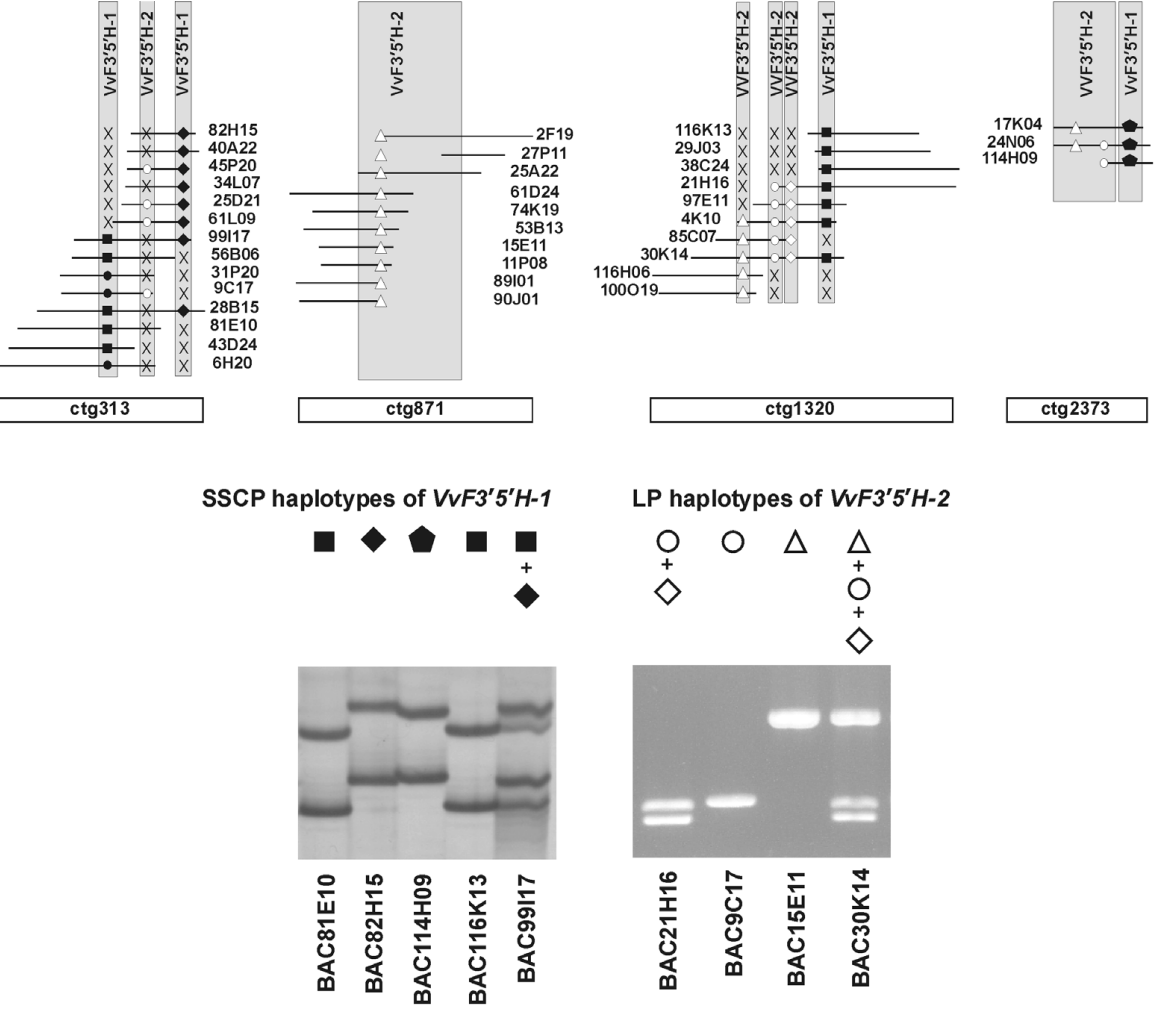
**Haplotypes at the *VvF3'5'H-1 *and *VvF3'5'H-2 *loci**. SSCP haplotypes at the *VvF3'5'H-1 *locus and length polymorphic haplotypes at the *VvF3'5'H-2 *locus were identified in the BAC clones positive for the presence of flavonoid 3',5'-hydroxylase genes on contigs ctg313, ctg871, ctg1320 and ctg2373 (*above*). *Names *written in horizontal refer to BAC clones and contigs; those written in vertical refer to gene copies of flavonoid 3',5'-hydroxylases. The relation between the *symbol *(■ ◆  ● ◇ ○ △) used in the picture and the corresponding haplotype displayed in a representative BAC clone is shown *below*. The symbol 'X' stands for lack of the presence of a given haplotype in the corresponding BAC clone.

Flavonoid 3',5'-hydroxylase genes were present in four contigs (ctg313, ctg871, ctg1320, and ctg2373) that covered approximately 450, 600, 280 and 140 kb, respectively. Each one of the contigs ctg313, ctg1320 and ctg2373 contained both *VvF3'5'H-1 *and *VvF3'5'H-2 *gene fragments. Contig ctg871 contained only the *VvF3'5'H-2 *gene fragment. Contigs ctg313, ctg871, ctg1320 and ctg2373 included 26, 29, 10 and 3 BAC clones, respectively, of which 14, 10, 10 and 3, respectively, contained either one or both isoforms of flavonoid 3',5'-hydroxylases as shown in Figure 6. *VvF3'5'H-1 *and *VvF3'5'H-2 *fragments were amplified from each individual positive BAC clone of contigs ctg313, ctg871, ctg1320 and ctg2373 using the primers pairs F35-1int and F35-2int, respectively, and analyzed by the SSCP technique.

Based on BAC order and the number of SSCP haplotypes, at a first approximation we hypothesize the occurrence of four loci containing *VvF3'5'H-1*-type sequences and seven loci containing *VvF3'5'H-2*-type sequences over the four contigs (Figure 6). Two paralogous copies of gene *VvF3'5'H-1 *were identified in ctg313. The first paralog had two allelic variants, that could be detected by SSCP analysis, one present in three BAC clones 6H20, 9C17, 31P20 (corresponding to the SSCP band genetically mapped from 'Cabernet Sauvignon') and the other in the remaining five clones (data not shown). Two BAC clones (28B15 and 99I17) spanned the region that included both paralogs. A third potential paralogous copy of the gene *VvF3'5'H-1 *was identified in contig ctg1320 whose BAC clones contained one of the two SSCP haplotypes present in the BACs from contig ctg313. An additional different haplotype of the gene *VvF3'5'H-1 *was identified in contig ctg2373 that included just three BAC clones. We could detect a potential of seven paralogous copies of the gene *VvF3'5'H-2 *in contigs ctg313, ctg871, ctg1320 and ctg2373. The primers pair F35-2int identified three types of gene fragments distinguishable by size, hereafter referred to as short, medium and long (Figure 6). All three gene fragments were found in ctg1320 in the close vicinity of the gene copy of *VvF3'5'H-1*. Three BAC clones contained all three forms at the same time, confirming the paralogous nature of these *VvF3'5'H-2 *gene copies. The medium sized gene fragment *VvF3'5'H-2 *was also amplified in ctg313 and ctg2373 where it showed a SSCP haplotype different from that found in ctg1320 (data not shown). A different locus carrying the longer fragment of *VvF3'5'H-2 *was present in ctg871 with a SSCP haplotype not distinguishable from that found in ctg1320.

### Integration of genetic and physical maps around the locus *VvF3'5'H*

**Figure 7:**
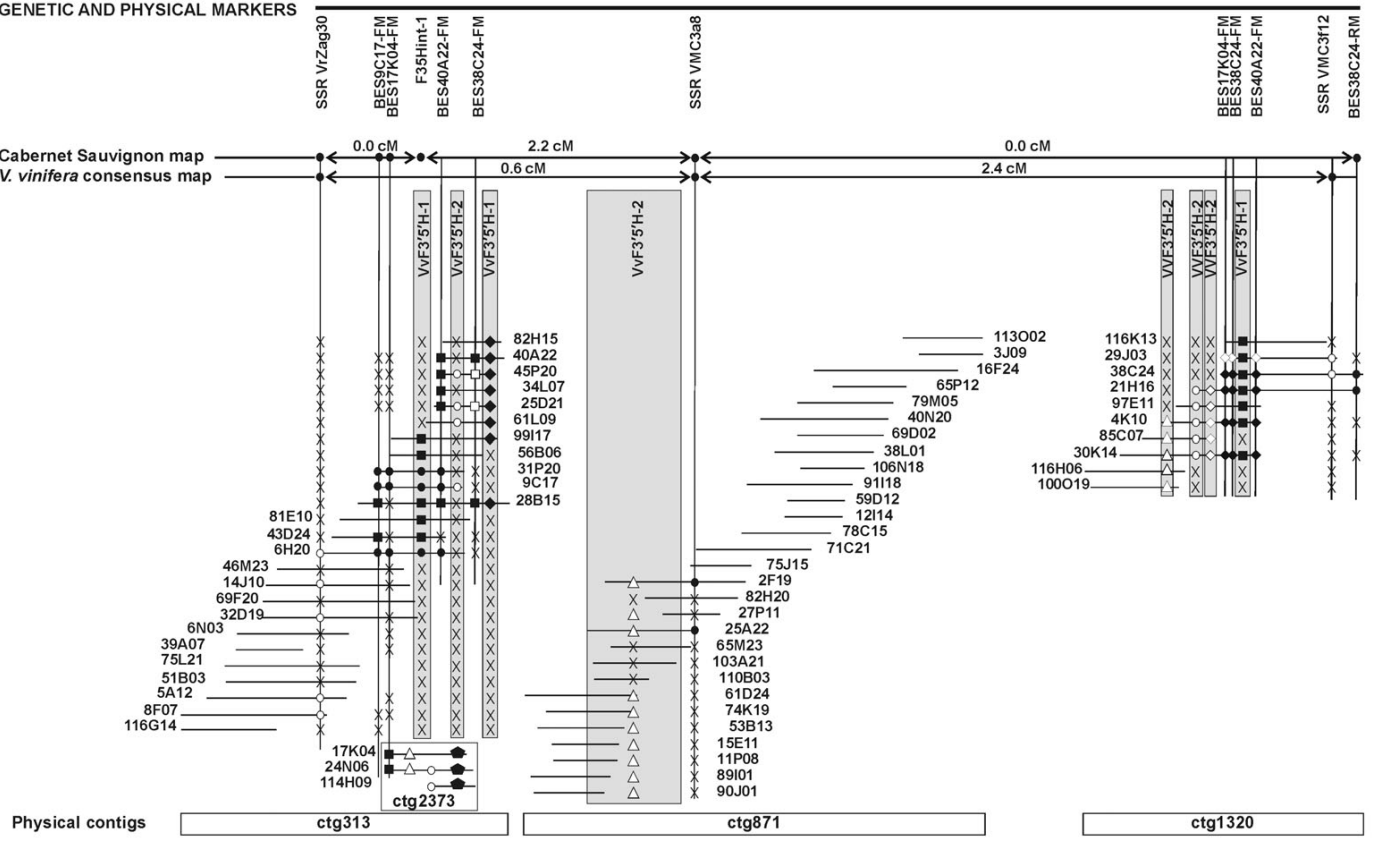
**Integrated genetic and local physical map spanning the complex locus *VvF3'5'H *on LG6**. Contigs were assembled using FPC and BAC clones were further aligned using BES-derived markers. Contigs were anchored to the 'Cabernet Sauvignon' and the *V. vinifera *consensus genetic maps using cross-referenced SSR markers. The orientation of each contig is arbitrary. Genetic and physical distances are not drawn to scale. *Names *written in horizontal refer to BAC clones and contigs; those written in vertical refer to markers and genes. The presence of a given marker on a BAC clone is highlighted with a symbol and different SSCP haplotypes for each marker are shown with different *symbols *(■ ◆  ● ◇ ○ △). The *symbol *'X' stands for lack of amplification of a given marker in the corresponding BAC clone. BAC clones missing any symbols were not tested with the corresponding marker. Physical localisation of *VvF3'5'H-1 *and *VvF3'5'H-2 *loci is shown in grey *boxes*.

Contigs ctg313, ctg871, ctg1320 and ctg2373 were anchored to the 'Cabernet Sauvignon' genetic map by using SSR markers present in the BAC clones and single strand conformational polymorphism of the BAC-end sequences (Figure 7). The SSR marker VrZag30 that was identified in five BACs of ctg313 allowed this contig to be anchored to LG6. Map position of ctg313 was confirmed by mapping the SSCP marker BES9C17-FM developed on a BAC clone embedded into ctg313 that co-segregated with SSR VrZag30. The contig ctg871 was anchored to LG6 by the SSR marker VMC3a8. The contig ctg1320 was anchored to LG6 using the SSR VMC3f12 and the BAC end-derived SSCP marker BES38C24-RM. The contig ctg2373 was anchored to LG6 by the BAC end-derived SSCP marker BES17K4-FM. The SSCP band of the marker BES17K04-FM inherited from 'Cabernet Sauvignon', which corresponded to the SSCP band amplified from the BAC clone 17K04, was amplified in the mapping population and completely co-segregated with SSR VrZag30 and BES9C17-FM. The marker BES38C24-RM mapped 2.2 cM apart from those three markers and co-segregated with SSR VMC3a8. All contigs ctg313, ctg871, ctg1320 and ctg2373 were located on LG6 in the region covered by the markers VrZag30, VMC3a8, BES9C17-FM, BES17K4-FM and BES38C24-RM. This interval spanned 2.2 cM in the 'Cabernet Sauvignon' parental map. Contigs ctg313, ctg871, ctg1320 were also cross-referenced to the *V. vinifera *consensus map [A. Doligez, unpublished data] by the SSR markers VrZag30, VMC3a8 and VMC3f12, respectively. The region spanned 3.0 cM and the median VMC3a8 marker was settled 0.6 cM and 2.4 cM away from VrZag30 and VMC3f12, respectively (Figure 7).

The BES of positive BAC clones provided new markers that were closely linked to the flavonoid 3',5'-hydroxylase gene copies and served to characterize that region more precisely. The marker BES9C17-FM was located on the physical map downstream of SSR VrZag30 and identified only BAC clones belonging to the contig ctg313. BES38C24-RM matched uniquely the BAC clones 38C24 and 21H16 belonging to ctg1320. Conversely, BES40A22-FM and BES38C24-FM primers were designed on BES belonging to ctg313 and ctg1320, respectively, but cross-identify BAC clones assembled in both contigs. SSCP analysis of BES40A22-FM and BES38C24-FM fragments revealed that for each marker two haplotypes are present in ctg313 and two different haplotypes are present in ctg1320. Yet, marker BES17K04-FM matched BACs 6H20, 9C17 and 31P20 of ctg313 and BACs 29J23, 38C24, 4K10, 30K14 of ctg1320. SSCP analysis identified four different haplotypes at the regions amplified by BES17K04-FM primers: two haplotypes were detected in the BAC clones of ctg1320, one more different haplotype was present in ctg313, and yet another SSCP haplotype was found in both BAC17K04 and BAC24N06 belonging to ctg2373. The occurrence of more than two haplotypes of the flavonoid 3',5'-hydroxylase gene fragments and of the BES markers in a diploid genotype confirmed the presence of paralogous copies of the flavonoid 3',5'-hydroxylase genes and rule out the possibility that the individual contigs represent allelic regions that have not been properly assembled. A segmental duplication spanning the markers F35-1int, F35-2int, BES40A22-FM, BES17K04-FM and BES38C24-FM that occurred within 2.2-3.0 cM on LG6 could explain the physical organization of flavonoid 3',5'-hydroxylase isogenes as it has been outlined in Figure 7. The contig ctg2373 that comprised only three BAC clones could not be unambiguously positioned in relation to the other contigs. BES markers from the other contigs did not match any BAC clones of ctg2373 but marker BES17K04-FM matched both ctg313 and ctg1320. Marker BES17K04-FM is therefore located within the segmental duplication. It remains uncertain whether ctg2373 partially overlaps the region of ctg313 and split from it due to high heterozygosity or if it actually covers an adjacent locus. Under the assumption of BAC alignment between BAC clones 9C17 and 17K04 that is supported by genetic co-segregation of the corresponding BES and by co-amplification by marker BES17K04-FM, ctg2373 would carry the haplotype allelic to BACs 6H20, 9C17, 31P20 at the BES17K04-FM SSCP marker and the *VvF3'5'H-1 *gene fragment present in BAC17K04 would miss a counterpart on the homologous chromosome. Otherwise, ctg2373 would identify a forth different locus of *VvF3'5'H-1*, located in the surrounding region and under-represented among the BAC clones of the library.

Based on the data available to us, we propose that the chromosomal region spanning the *VvF3'5H *gene cluster includes four copies of *VvF3'5'H-1 *and five to seven copies of *VvF3'5'H-2*, all sharing a high sequence similarity. We were able to present a preliminary physical map of this cluster of genes by a combination of computational assembly of fingerprinted BACs and by PCR-based alignment of BAC clones. The region has likely originated from both tandem duplications that generated nearby gene copies (i.e. paralogs within BACs 28B15 and 99I17 in the ctg313) and from segmental intrachromosomal duplications involving a larger block of DNA (i.e. blocks of paralogs duplicated into ctg313 and ctg1320). Segmental duplications are an important source of gene evolution [25] and they have been estimated to cover 1.7–2.0% of the mouse genome [26] and 4% of the human genome [27]. In the rat genome, duplicated genes are largely confined into <1 Mb intrachromosomal duplications rather than widely interspersed through the genome [28]. A similar model of genome dynamics could also have shaped plant genomes as it is emerging for model species [29].

### Expression analysis of *VvF3'H *and *VvF3'5'H *along with major structural genes of the flavonoid pathway in different grape tissues

**Figure 8:**
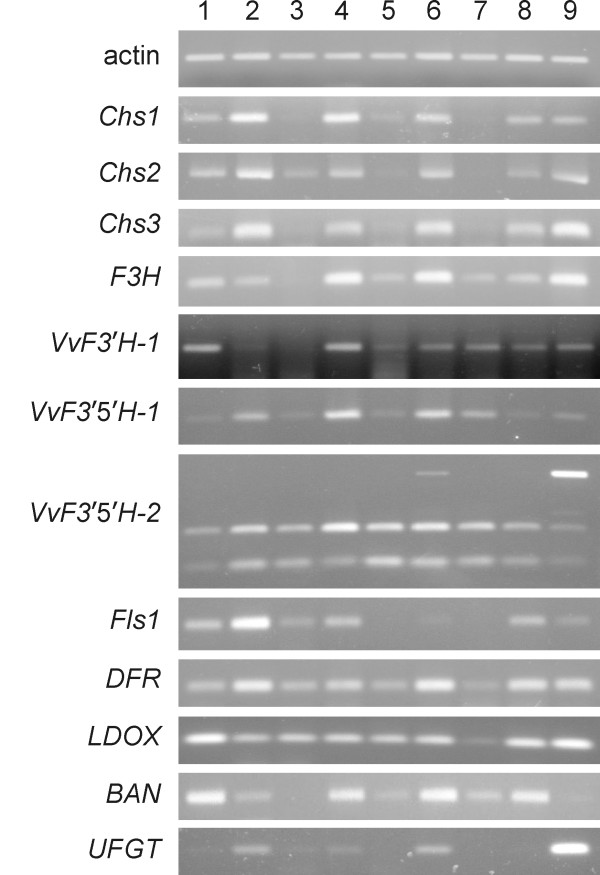
**Expression of structural genes of the flavonoid pathway in different tissues**. Expression profiles of flavonoid hydroxylase genes were analysed alongside nine major structural genes of the flavonoid pathway in the following tissues: 1, apex and apical leaflet (pale green); 2, apex and apical leaflets (reddish pigmented shoot tips); 3, fully expanded leaves; 4, immature flowers; 5, rootlets; 6, seeds; 7, berry flesh; 8, berry skin (pre-*véraison*, green); 9, berry skin (post-*véraison*, red). The actin gene was used as a constitutive gene for the normalisation of cDNA samples.

The genes encoding both flavonoid 3'-hydroxylase and flavonoid 3'-5'-hydroxylases are widely expressed in grapevine organs (Figure 8). Transcripts of gene *VvF3'H-1 *were detected in all tissues tested. *VvF3'H-1 *was strongly expressed in green apical leaflets and flowers but it was only slightly detectable in fully expanded leaves. It was also present in all berry tissues. The other sequence *VvF3'H-2*, which translated into a truncated flavonoid 3'-hydroxylase due to a 23-bp frameshift deletion, was apparently not expressed and is likely to be a pseudogene.

Transcripts of *VvF3'5'H-1 *were present in all tissues. High levels of expression were detected in pigmented apical leaflets, flowers, seeds and berries but not in green fully expanded leaves and roots. Differential expression could be detected between green berry skin and red berry skin, the expression being higher in pigmented berry skin. The expression pattern of *VvF3'5'H-1 *mimicked the expression profile of *Chs2*, *Chs3 *and the gene coding for flavanone hydroxylase (*F3H*). The gene *VvF3'5'H-2 *expressed transcripts of different size that could be distinguished on agarose gel (*VvF3'5'H-2*-short, *VvF3'5'H-2*-medium and *VvF3'5'H-2*-long). The two shorter bands were invariantly present in all tissues, even though at a different level of expression. The longest band was strongly expressed only in red berry skin, whilst it was slightly detectable in seeds and not detectable at all in other tissues.

The expression of most flavonoid genes, including those coding for flavonoid hydroxylases, was more intensively activated in flowers and seeds than in other organs. Seeds expressed all isoforms of the flavonoid hydroxylase genes identified in this work, whilst all but *VvF3'5'H-2*-long were expressed in flowers. Among berry tissues, flesh poorly expressed the genes of the flavonoid pathway, except for *BAN*, *VvF3'H-1*, *VvF3'5'H-1*, *VvF3'5'H-2*-short and *VvF3'5'H-2*-medium. In the epicarp of berries collected at different developmental stages, *Fls1 *and *BAN *were more expressed at the pre-*véraison *stage; *Chs3*, *F3H *and *VvF3'5'H-1 *were induced in red skin compared to green skin; *UFGT*, *VvF3'H-1 *and *VvF3'5'H-2*-long were strongly expressed in red skin but were undetectable in green skin.

**Figure 9:**
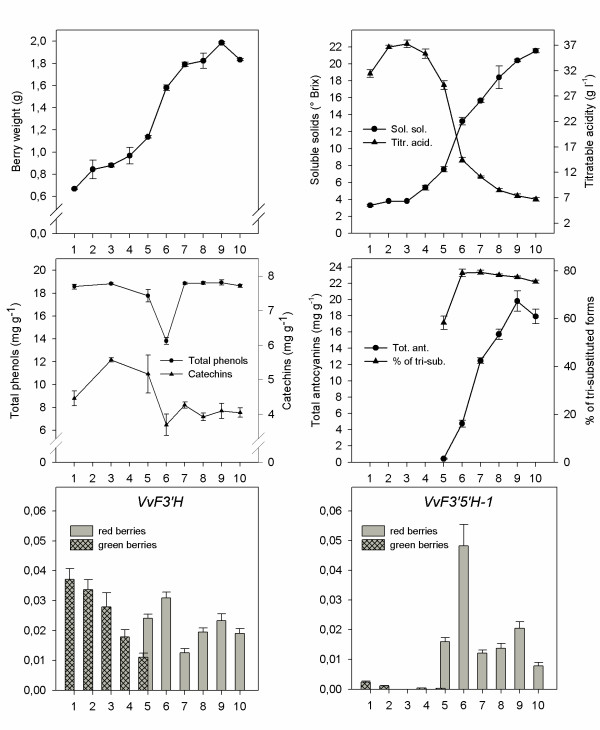
**Transcript profiling of *VvF3'H *and *VvF3'5'H-1 *in ripening berries**. Ripening curve of *V. vinifera *'Merlot' was based on analytical parameters (berry weight, soluble solids, titratable acidity expressed as tartaric acid equivalents, skin total phenols expressed as (+)-gallic acid, skin catechins expressed as (+)-catechin, skin anthocyanins) at the following sampling dates: 1. July 15^th^, 2. July 29^th^, 3. August 3^rd^, 4. August 9^th^, 5. August 12^th^, 6. August 24^th^, 7. September 3^rd^, 8. September 13^th^, 9. September 20^th^, 10. September 28^th^. Concomitant gene expression of *VvF3'H*-1 and *VvF3'5'H-1 *in berry skin was assessed by quantitative RT-PCR and expressed as the ratio between the C_T _of the gene under study and the C_T _of the actin gene. Bars represent ± s.e..

The flavonoid pathway does not exclusively lead to the synthesis of anthocyanins and several flavonoid hydroxylase isogenes were therefore expressed also in non-pigmented tissues. Berry flesh and seeds accumulate non-anthocyanin flavonoids such as proanthocyanidins and catechins. Their synthesis is catalysed downstream of the flavonoid hydroxylases by the genes *DFR*, *LDOX *and *BAN*. Gene *BAN *was strongly expressed in seeds, berry flesh and berry skin at pre-*véraison*, whilst was almost undetectable in post-*véraison *berry skin at the stage when the rate of accumulation of proanthocyanins and catechins is declining (Figure 9). Sunlight exposed tissues expressed the gene coding for flavonol synthase (*Fls1*). Flavonol synthase converts the products of flavonoid hydroxylases into the UV-protecting flavonols. Inner tissues (seeds and berry flesh) and underground organs (rootlets) did not exhibit detectable levels of *Fls1*.

### Expression profiling of flavonoid hydroxylase genes in skin of ripening berries

The time-course of expression of flavonoid hydroxylase genes in the red-skinned cultivar 'Merlot' revealed that gene *VvF3'H-1 *is expressed throughout ripening. High transcript levels were detected from the first sampling date at the stage when catechins are rapidly synthesised and peak at 5.6 mg g^-1 ^of berry skin (Figure 9). *VvF3'H-1 *expression decreased 8–10 weeks after blooming, at the ripening stage when most genes committed to the flavonoid pathway have been reported to level off [1]. *VvF3'H-1 *transcripts increased soon after, at the onset of *véraison *(August 12^th^), which were at that point in time 2-fold higher in red berries than in green berries collected from the same bunch. *VvF3'H-1 *expression lasted till harvest, with a secondary peak of expression one week before harvest, concurrently at the peak of anthocyanin content.

**Figure 10:**
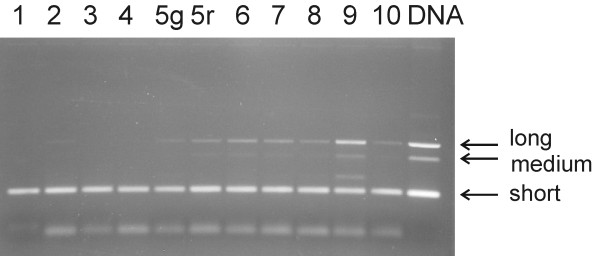
**Expression of *VvF3'5'H-2 *during the progress of ripening**. Length polymorphic transcripts of *VvF3'5'H-2 *were detected semi-quantitatively after cDNA samples had been normalised upon actin gene expression, at the sampling dates reported in Fig 9. The short, medium and long transcripts were named according to the fragment size amplified from genomic DNA. At sampling 5, green berries (5g) were kept separated from red berries (5r). PCR amplification on 'Merlot' genomic DNA was loaded in the right extreme lane.

The gene *VvF3'5'H-1 *was weakly expressed during the earliest ripening stages. At the onset of *véraison*, transcripts of *VvF3'5'H-1 *were poorly detectable in green berries, while they increased 50-fold in berries that had already turned red within the same bunch. The peak of *VvF3'5'H-1 *expression corresponded to August 24^th^, at the stage when all berries had turned red. As described for *VvF3'H-1*, a weaker peak of expression was found one week before harvest. Expression of *VvF3'5'H-2 *was checked by semi-quantitative PCR because the different isoforms of this gene were distinguishable by size (Figure 10). Transcripts of the isoforms *VvF3'5'H-2*-long and *VvF3'5'H-2*-medium did appear at the onset of *véraison *and lasted till full ripening, whilst the isogene *VvF3'5'H-2*-short was constitutively expressed throughout the ripening.

### Accumulation, composition of anthocyanins and colour evolution in berry skin during the progress of ripening

Biometric and analytical parameters have been used to follow the progress of ripening (Figure 9). The onset of ripening began on August 3^rd ^when sugars started to accumulate and acidity commenced to decline. The onset of *véraison *(August 12^th^) was considered as the time when 10% of the berries had turned red (*H*° = 102.98, C* = 18.27). Bunches went through this phase of colour transition until August 24^th ^when all berries had turned red (*H*° = 20.46, C* = 7.43). Anthocyanins have been detected by HPLC analysis since the onset of *véraison *on August 12^th ^(0.4 mg g^-1^). The accumulation of anthocyanins lasted until September 20^th^, one week before harvest (19.8 mg g^-1^), slightly decreasing at harvest (17.1 mg g^-1^).

At the earliest stage of the *véraison*, cyanidin-based anthocyanins (cyanidin and peonidin) were roughly as abundant as delphinidin-based anthocyanins (delphinidin, malvidin and petunidin) (42% versus 58%). Then, on August 24^th ^the percentage of delphinidin-based anthocyanin (79%) peaked simultaneously with the highest level of expression of *VvF3'5'H-1*. The fraction of tri-substituted anthocyanins slightly decreased in the following weeks (77–78%), then remaining substantially unchanged until harvest (75%). Berry skin colour moderately varied from *H*° = 331.55, C* = 3.83 to *H*° = 306, C* = 2.59 from September 2^nd ^to September 28^th^. Anthocyanin composition in berry skin changed over the period of *véraison*, from the onset of colour transition till whole-bunch pigmentation, but remained roughly unmodified throughout the last four weeks of ripening. Although the anthocyanin profile of 'Merlot' was largely composed by malvidin and tri-substituted anthocyanins at harvest, derivatives of cyanidin were more promptly synthesised at the very early stage of *véraison*. The pattern of expression of *VvF3'H-1*, *VvF3'5'H-1 *and *VvF3'5'H-2 *is thus compatible with the hypothesis that they are strongly involved in the synthesis of the different types of anthocyanins and they affect anthocyanin composition in berry skin.

## Conclusion

We have shown that genes coding for flavonoid 3'- and 3',5'-hydroxylases are present as low/medium copy members in the grapevine genome and are organised in complex gene clusters that most likely resulted from gene duplication and intra-chromosomal segmental duplication. These genes are expressed in coordination with other genes of the flavonoid pathway in any tissues of the grape plant that are known to accumulate flavonoids, such as leaves, flowers, roots, seeds and, particularly, in skin of ripening berries. RT-PCR showed a strong relationship between the expression of *VvF3'H-1 *and *VvF3'5'H *and the kinetics of accumulation of cyanidin- and delphinidin-based anthocyanins in the red-skinned variety 'Merlot'. The presence of multiple putative 3',5'-hydroxylase isogenes which emerged from fine structural analysis at physical level, has first been shown for plant hydroxylases. This complexity was not unexpected for species like grape which synthesize large amounts of different classes of polyphenols and display a continuous variation of berry skin colour from brick red to dark blue among the so called red-skinned cultivars.

## Methods

### Plant material

Genomic DNA was extracted from young leaves [30]. 'Cabernet Sauvignon' was used for cloning flavonoid 3'- and 3',5'-hydroxylase genes. Forty six full-sibs of the cross 'Cabernet Sauvignon' × *Vitis *'breeding line 20/3' were used for genetic mapping. Gene expression was analysed in the red variety 'Merlot'. Apical leaflets of 1 cm^2 ^surface area, fully expanded leaves (>10 cm^2^) and adventitious rootlets were collected from canes grown in hydroponic culture for two months. Flower buttons were collected one week before blooming, seeds and flesh were extracted from berries collected two weeks after *véraison*. Transcript and metabolite profiling in berry skin was performed on samples of 40 berries collected at ten ripening stages from pre-*véraison *(July 15^th^, 2004) till harvest (September 28^th^, 2004) from plots of twelve vines replicated four times.

### Cloning of flavonoid 3'- and 3',5'-hydroxylase genes with the help of degenerate primers

Degenerate primers were designed on two specific and conserved regions of plant F3'H and F3',5'H genes (Table 1, Figure 1). PCRs were performed in a 40 μl volume containing 200 μM of each dNTP, 2.5 μM of each primer, 1 U of HotMaster *Taq *polymerase (Eppendorf) with 2.5 mM MgCl_2 _buffer, and 40 ng of template DNA. The denaturation step was 95°C for 1 min, followed by 40 cycles of 92°C for 45 s, 60°C for F3'H primers and 56°C for F3',5'H primers for 50 s, 65°C for 90 s. Amplified bands were extracted from agarose gel with Nucleospin Extract (Macherey-Nagel) and cloned into pGEM-T vector (Promega). Unique clones were identified by means of PCR with M13 universal primers and SSCP-fingerprinting of the insert. Selected clones were sequenced with BigDye terminator chemistry and a MegaBace500 sequencer (Amersham Biosciences).

### Sequence analysis

Nucleotide and amino acid sequences were compared with GenBank accessions, with EST singletons and tentative consensus sequences (TCs) held at the TIGR grape gene index. Conceptual translation was obtained with DNAclub software. Amino acid entries of 670 plant cytochrome P450 monooxygenases were downloaded from GenBank as of November 30^th^, 2004. Cluster analysis of plant cyt P450s and the candidate grape flavonoid hydroxylases was done with the Neighbor-joining method.

### Genetic mapping

Nested primers were designed to amplify an exon of grapevine flavonoid 3'- and 3',5'-hydroxylase genes (F3, F3-1, F35-1, F35-2, see Table 1 and 2). Additional primer pairs (F3int, F35-1int and F35-2int) were designed in the exon sequences flanking an intron for detecting SNPs useful for mapping in untranslated regions (Table 1 and 2). Flavonoid hydroxylase gene fragments containing the intron were amplified in a 10 μl volume containing 200 μM of dNTPs, 0.4 μM of each primer, 0.5 U of *Taq *polymerase, 2.5 mM MgCl_2 _and 20 ng of template DNA. The PCR profile was 35 cycles of 92°C for 50s, annealing temperature as reported in Table 2 for 50s, 65°C for 1 min. Gene fragments were separated on 4% glycerol and 0.5 × MDE polyacrylamide gel (BioWhittaker Molecular Applications) for SSCP analysis, run at room temperature for 16 hours at 6 W and stained with silver nitrate. The segregation of F3int, F35-1int, F35-2int and 335 SSR markers was followed in the 'Cabernet Sauvignon' × *Vitis *'breeding line 20/3' full-sib family [G. Di Gaspero, unpublished data]. Linkage groups of markers segregating from the 'Cabernet Sauvignon' parent were identified using Carthagene with a LOD of 5.0 [31]. Marker order was calculated using the command Build 3.

### Local physical maps

A 6X BAC library of 'Cabernet Sauvignon' was screened by PCR with the F3, F35-1 and F35-2 primer pairs (Table 2) using a 3D pooling strategy [23]. Then, positive individual clones were pre-cultured in 2X LB medium for 20 hours and 2 μL of cell suspension were inoculated into the same medium and grown for 16 hours. Cultured clones were directly used as PCR template. Positive BACs were confirmed using the corresponding F3int, F35-1int and F35-2int primer pairs (Table 2). PCR products were then run into a SSCP gel (as above) for identifying the haplotype of each BAC clone. A set of 30,151 BAC fingerprints of the 'Cabernet Sauvignon' BAC clones [S. Paillard, unpublished data] were assembled into contigs using FPC v8.0 [24]. The first assembly was made at a cut-off value of 10^-50 ^and with a tolerance of 0.4 bp. After several steps of dQing and merging, 2,035 BAC contigs were obtained [S. Paillard, unpublished data]. BAC clones that were positive at the PCR screening were localized over the contigs. Clone order was determined by running the consensus band (CB) algorithm on each individual positive contig. Contig size was estimated by converting CB units into physical length, under the assumption of a band mean size of 1,100 bp. Positive contigs were anchored to the genetic map by means of already mapped SSR markers that were present in the assembled BACs [[23], Adam-Blondon, unpublished data]. Further SSCP markers (38C24-RM, 17K14-FM, 9C17-FM, 40A22-FM and 5A23-FM, Table 1 and 2) were developed on the BAC-end sequences (BES) [A.F. Adam-Blondon and S. Paillard, unpublished data] of the positive clones and enabled new genetic markers to be identified that were closely linked to *VvF3'H *and *VvF3'5'H *loci (Table 1 and 2). BES-derived markers (Table 1 and 2) were also used to cross-reference the BAC clones within a contig, validate the clone order and identify the BAC haplotypes in regions where allelic BACs were incorporated into a given contig.

### Transcript profiling

Total RNA was extracted from berry skin and flesh, leaves, flower bunches, rootlets and seeds following the procedure described in [31]. Total RNA was treated with 0.5 U/μg RQ1 DNase (Promega) in presence of an RNAse inhibitor (RNAguard, Amersham Biosciences). First strand cDNA was synthesised using 2 μg of RNA, 0.5 μM (dT)_18 _primer and 50 U of M-MLV Reverse Transcriptase (Promega). Quantitative real-time PCR was carried out on a DNA Engine Opticon2 (MJ Research) using SYBR Green Jump Start Taq ReadyMix (Sigma). The primer pairs F3-1, F35-1 and F35-2 (Table 2) were used for expression analysis of *VvF3'H-1*, *VvF3'5'H-1 *and *VvF3'5'H-2*, respectively. Each cDNA sample was analysed at two different dilutions and with two replicates per dilution. Gene transcripts were quantified comparing the threshold cycle (C_T_) with that of the constitutive actin gene [32]. Semi-quantitative PCR was performed on different tissues upon cDNA normalisation based on the expression of the actin gene and visualised on agarose gel stained with ethidium bromide. Primers pairs for the genes of the flavonoid pathway were retrieved from the literature: primers for *Chs1*, *Chs2*, *Chs3*, *DFR*, *LDOX*, *UFGT *were from [33], primers for *Fls1 *were from [34]. Primers for *F3H *and *BAN *were newly designed on the original gene sequences [35,36] to tag 150–200 bp gene fragments.

### Metabolite profiling during berry development

Total soluble solids were quantified with a refractometer (Analytical control) and expressed as °Brix. Titratable acidity was measured with an automated titrator (Crison) and expressed as tartaric acid equivalents. Skin phenols were extracted for 4 hours in 1/10 (w/v) skin/solvent suspension of 100% methanol and 1% HCl, then centrifuged at 5,000 rpm for 15 min and filtered with a 0.2 μm PTFE filter (Chemtek Analytica). Total phenols were determined following the procedure of [37]. The calibration curve was obtained with (+)-gallic acid (Sigma). Total flavan-3-ols (catechins) were quantified as reported in [38]. The calibration curve was constructed with (+)-catechin (Extrasynthase). Absorbance was measured by a UV/VIS LAMBDA 2 spectrometer (Perkin-Elmer). Anthocyanins for the HPLC analysis were extracted for 4 hours from 200 mg of berry skin with 2 mL of methanol, then centrifuged and filtered with a 0.2 μm PTFE filter (Chemtek analitica). Methanol was evaporated under nitrogen flux and anthocyanins were re-suspended with 100–400 μL of 27:73 methanol:perchloric acid 0.3% (v/v). Anthocyanins were separated in a high performance liquid chromatograph (Perkin Elmer Series 4) using a C18 Purospher RP-18 (5 μm, 250 mm × 4 mm) column (Merck) protected by a C18 guard column, according to the procedure reported in [39]. Anthocyanins were detected by a LC-95 spectrophotometric detector (Perkin Elmer) operating at 520 nm. Anthocyanin content was expressed as mg/L of malvidin 3-glucoside upon the construction of a standard curve. The composition of monoglucoside anthocyanins was used for calculating the ratio of tri-/di-substituted derivatives. Berry colour was measured with an X-Rite 948 Chromameter (X-Rite) and averaged over 160 berries at each sampling date. Colorimetric specification was referenced to the CIELab scale. Hue angle (*H*°) and chroma (C*) were calculated according to [40].

## Authors' contributions

SDC participated in the design of the study, carried out gene cloning, expression profiling, field experiments and analyses of metabolites, participated in the interpretation of the results. GDG conceived the study, coordinated the experiments, carried out genetic mapping, interpreted the results and drafted the manuscript. RM managed and screened the BAC library. AN carried out and interpreted the expression analysis. EP critically revised the manuscript. SP constructed the local physical maps and participated in the interpretation of those results. AFAB coordinated the construction of the physical map and critically revised the manuscript. RT revised the manuscript for important intellectual content. All authors read and approved the final manuscript.

## References

[B1] Boss PK, Davis C, Robinson SP (1996). Analysis of the expression of anthocyanin pathway genes in developing *Vitis vinifera *L. cv Shiraz grape berries and the implications for pathway regulation. Plant Physiol.

[B2] Kobayashi S, Ishimaru M, Ding CK, Yakushiji H, Goto N (2001). Comparison of UDP-glucose:flavonoid 3-*O*-glucosyltransferase (UFGT) gene sequences between white grapes (*Vitis vinifera*) and their spots with red skin. Plant Sci.

[B3] Kobayashi S, Ishimaru M, Hiraoka K, Honda C (2002). *Myb*-related genes of the Kyoho grape (*Vitis labruscana*) regulate anthocyanin biosynthesis. Planta.

[B4] Ford CM, Boss PK, Høi PB (1998). Cloning and characterization of *Vitis vinifera *UDP-glucose:flavonoid 3-*O*-glucosyltransferase, a homologue of the enzyme encoded by the maize *Bronze-1 *locus that may primarily serve to glucosylate anthocyanidins *in vivo*. J Biol Chem.

[B5] Holton AT, Cornish EC (1995). Genetics and biochemistry of anthocyanin biosynthesis. Plant Cell.

[B6] Jaakola L, Määttä K, Pirttilä AM, Törrönen R, Kärenlampi S, Hohtola A (2002). Expression of genes involved in anthocyanin biosynthesis in relation to anthocyanin, proanthocyanidin, and flavonol levels during bilberry fruit development. Plant Physiol.

[B7] Albach RF, Kepner RE, Webb AD (1959). Comparison of anthocyanin pigments of red vinifera grapes. Am J Enol Vitic.

[B8] Roggero JP, Coen S, Larice JL (1986). Etude comparative de la composition anthocyanique des cepages. Bull Liais Groupe Polyph.

[B9] Esteban MA, Villanueva MJ, Lissarague JR (2001). Effect of irrigation on changes in anthocyanin composition of the skin of cv Tempranillo (*Vitis vinifera *L.) grape berries during ripening. J Sci Food Agr.

[B10] Spayd SE, Tarara JM, Mee DL, Fergurson JC (2002). Separation of sunlight and temperature effect on the composition of *Vitis vinifera *cv. Merlot berries. Am J Enol Vitic.

[B11] Martin C, Prescott A, Mackay S, Bartlett J, Vrijlandt E (1991). Control of anthocyanin biosynthesis in flowers of *Antirrhinum majus*. Plant J.

[B12] Okinaka Y, Shimada Y, Nakano-Shimada R, Ohbayashi M, Kiyokawa S, Kikuchi Y (2003). Selective accumulation of delphinidin derivatives in tobacco using a putative flavonoid 3',5'-hydroxylase cDNA from *Campanula medium*. Biosci Biotech Bioch.

[B13] Fukui Y, Tanaka Y, Kusumi T, Iwashita T, Nomoto K (2003). A rationale for the shift in colour towards blue in transgenic carnation flowers expressing the flavonoid 3',5'-hydroxylase gene. Phytochemistry.

[B14] Tanaka Y, Yonekura K, Fukuchi-Mizutani M, Fukui Y, Fujiwara H, Ashikari T, Kusumi T (1996). Molecular and biochemical characterization of three anthocyanin synthetic enzymes from *Gentiana triflora*. Plant Cell Physiol.

[B15] Zufall RA, Rausher MD (2003). The genetic basis of a flower colour polymorphism in the common morning glory (*Ipomoea purpurea*). Heredity.

[B16] Brugliera F, Barri-Rewell G, Holton TA, Mason JG (1999). Isolation and characterization of a flavonoid 3'-hydroxylase cDNA clone corresponding to the Ht1 locus of *Petunia hybrida*. Plant J.

[B17] Su V, Hsu BD (2003). Cloning and expression of a putative cytochrome P450 gene that influences the colour of *Phalaenopsis flowers*. Biotechnol Lett.

[B18] Mori S, Kobayashi H, Hoshi Y, Kondo M, Nakano M (2004). Heterologous expression of the flavonoid 3',5'-hydroxylase gene of *Vinca major *alters flower colour in transgenic *Petunia hybrida*. Plant Cell Rep.

[B19] Paquette SM, Bak S, Feyereisen R (2000). Intron-exon organization and phylogeny in a large superfamily, the paralogous cytochrome P450 Genes of *Arabidopsis thaliana*. DNA Cell Biol.

[B20] Nelson DR, Schuler MA, Paquette SM, Werck-Reichhart D, Bak S (2004). Comparative genomics of rice and *Arabidopsis*. Analysis of 727 cytochrome P450 genes and pseudogenes from a monocot and a dicot. Plant Physiol.

[B21] Schoenbohm C, Martens S, Eder C, Forkmann G, Weisshaar B (2000). Identification of the *Arabidopsis thaliana *flavonoid 3'-hydroxylase gene and functional expression of the encoded P450 enzyme. Biol Chem.

[B22] Riaz S, Dangl GS, Edwards KJ, Meredith CP (2004). A microsatellite marker based framework linkage map of *Vitis vinifera *L. Theor Appl Genet.

[B23] Adam-Blondon AF, Bernole A, Faes G, Lamoureux D, Pateyron S, Grando MS, Caboche M, Velasco R, Chalhoub B (2005). Construction and characterization of BAC libraries from major grapevine cultivars. Theor Appl Genet.

[B24] Soderlund C, Humphray S, Dunham A, French L (2000). Contigs built with fingerprints, markers, and FPC V4.7. Genome Res.

[B25] Ohno S (1970). Evolution by gene duplication.

[B26] Bailey JA, Church DM, Ventura M, Rocchi M, Eichler EE (2004). Analysis of segmental duplications and genome assembly in the mouse. Genome Res.

[B27] Zhang L, Lu HH, Chung WY, Yang J, Li WH (2005). Patterns of segmental duplication in the human genome. Mol Biol Evol.

[B28] Tuzun E, Bailey JA, Eichler EE (2004). Recent segmental duplications in the working draft assembly of the brown Norway rat. Genome Res.

[B29] Cannon SB, Mitra A, Baumgarten A, Young ND, May G (2004). The roles of segmental and tandem gene duplication in the evolution of large gene families in *Arabidopsis thaliana*. BMC Plant Biol.

[B30] Doyle JJ, Doyle JL (1990). Isolation of plant DNA from fresh tissue. Focus.

[B31] de Givry S, Bouchez M, Chabrier P, Milan D, Schiex T (2005). Carthagene: multipopulation integrated genetic and radiation hybrid mapping. Bioinformatics.

[B32] Moser C, Gatto P, Moser M, Pindo M, Velasco R (2004). Isolation of functional RNA from small amounts of different grape and apple tissues. Mol Biotechnol.

[B33] Livak KJ, Schmittgen TD (2001). Analysis of relative gene expression data using real-time quantitative PCR and the 2^-ΔΔC^_T _method. Methods.

[B34] Goto-Yamamoto N, Wan GH, Masaki K, Kobayashi S (2002). Structure and transcription of three chalcone synthase genes of grapevine (*Vitis vinifera*). Plant Sci.

[B35] Downey M, Harvey J, Robinson S (2003). Synthesis of flavonols and expression of flavonol synthase genes in developing grape berries of Shiraz and Chardonnay (*Vitis vinifera *L.). Aust J Grape Wine Res.

[B36] Sparvoli F, Martin C, Scienza A, Gavazzi G, Tonelli C (1994). Cloning and molecular analysis of structural genes involved in flavonoid and stilbene biosynthesis in grape (*Vitis vinifera *L.). Plant Mol Biol.

[B37] Tanner GJ, Francki KT, Abrahams S, Watson JM, Larkin PJ, Ashton AR (2003). Proanthocyanidin biosynthesis in plants: Purification of legume leucoanthocyanidin reductase and molecular cloning of its cDNA. J Biol Chem.

[B38] Singleton VL, Rossi JA (1965). Colorimetry of total phenolics with phosphomolybdic-phosphotungstic acid reagents. Am J Enol Vitic.

[B39] Zironi R, Buiatti S, Celotti E (1992). Evaluation of a new colorimetric method for the determination of catechins in musts and wines. Die Wein-Wissen.

[B40] Mattivi F, Failla O, Magliaretta L (1998). I pigmenti antocianici della bacca nella chemiotassonomia della vite. Girolamo Molon – L'Ampelografia e la Pomologia.

[B41] Gonnet JF (1998). Colour effects of co-pigmentation of anthocyanins revisited – 1. A colorimetric definition using the CIELAB scale. Food Chem.

[B42] Holton TA, Brugliera F, Lester DR, Tanaka Y, Hyland CD, Menting JG, Lu CY, Farcy E, Stevenson TW, Cornish EC (1993). Cloning and expression of cytochrome P450 genes controlling flower colour. Nature.

